# Variable Creatinine Levels in Critical Care Patients: A Concerning Knowledge Gap

**DOI:** 10.3390/jcm10081689

**Published:** 2021-04-15

**Authors:** Trushil Shah, Madhusudhanan Narasimhan, Mary Latha Rathinam, Karen Relle, Melanie Kim, Tharani Muthukumar, William Tharpe, Sonja Bartolome, Lenin Mahimainathan, Alagarraju Muthukumar

**Affiliations:** 1 Department of Internal Medicine, Pulmonary and Critical Care Medicine, University of Texas Southwestern Medical Center, Dallas, TX 75390, USA; trushil.shah@utsouthwestern.edu (T.S.); sonja.bartolome@utsouthwestern.edu (S.B.); 2 William P. Clements Jr. University Hospital (CUH), University of Texas Southwestern Medical Center, Dallas, TX 75235, USA; marylatha.rathinam@utsouthwestern.edu (M.L.R.); karen.relle@utsouthwestern.edu (K.R.); melanie.kim@utsouthwestern.edu (M.K.); william.tharpe@utsouthwestern.edu (W.T.); 3 Department of Pathology, University of Texas Southwestern Medical Center, Dallas, TX 75390, USA; madhusudhanan.narasimhan@utsouthwestern.edu (M.N.); lenin.mahimainathan@utsouthwestern.edu (L.M.); 4 School of Behavioral and Brain Sciences, University of Texas at Dallas, Richardson, TX 75080, USA; tharani.muthukumar@utdallas.edu

**Keywords:** creatinine enzymatic assay, interference, dopamine, dobutamine, awareness, survey

## Abstract

An accurate creatinine (Cr) estimate is pivotal for the assessment of renal function. Both patient- and practice-spawned factors palliate the test accuracy of serum creatinine (sCr) and can erratically represent actual kidney function. This study evaluated the caregivers’ awareness of enzymatic serum creatinine (E-sCr) assay interfering in dopamine/dobutamine (DD)-infused patient samples and the frequency of such interference in a critical care setting. We conducted an sCr awareness survey among UT Southwestern physicians, nurses, and pharmacists. We then performed a cross-sectional E-sCr comparison against the kinetic Jaffe method using the DD-infused patient samples collected from central venous catheters (CVC), peripherally inserted central catheter (PICC) lines, and the peripheral vein (PV). We retrospectively compared the longitudinal E-sCr results of the CVC/PICC draws with the corresponding blood urea nitrogen (BUN) levels. The survey results show a significant lack of awareness among caregivers about the negative interference of DD infusions on E-sCr. Cross-sectional E-sCr assessment relative to the Jaffe method displayed a negative interference in 12% of CVC/PICC line samples (7/57 DD-infused patients) compared to none in the PV draws. A longitudinal assessment of E-sCr, BUN, and potassium (K) levels from CVC/PICC line samples further confirmed a spurious decrease for E-sCr in about 12/50 (24%) patients who did not show a concurrent BUN or K decrease. The results suggest that a direct PV sampling accompanied by clinical laboratory-directed proactive discussion/activities can foster awareness among caregivers and eschew the false E-sCr estimates in DD-infused patients.

## 1. Introduction

Serum creatinine (sCr) is the standard of care marker for the assessment of renal function and serves as a strong independent risk factor for hospitalized patients and intensive care unit (ICU) mortality [1,2,3]. A slight perturbation in sCr levels is associated with significant in-hospital mortality, length of stay (LOS), and excess healthcare costs across a range of factors such as age, gender, diagnosis-related group weight, chronic kidney disease (CKD), and ICD-9 CM group codes [3]. It is worth noting that the International Society of Nephrology guideline defines and stages acute kidney injury (AKI) using the rise in sCr levels [4]. Thus, an accurate Cr estimate is pivotal for the assessment of renal function, the grouping of AKI, and classifying CKD appropriately. Despite its susceptibility to non-specificity issues, the Jaffe method remains the most popular and high volume test to estimate sCr because of its cost-effectiveness. The reaction involves measuring the change in absorption intensity due to the formation of a creatinine-picrate complex at an alkaline pH. Unfortunately, this method is fraught with analytical interference because of the presence of substances such as glucose, proteins, bilirubin, acetoacetate, cytosine, and drugs of the cephalosporin group in the samples [5]. This faulty measurement can not only misrepresent the actual functionality of the organ and lead to misdiagnosis, but also drive undesirable treatment outcomes, redundant laboratory testing, and unnecessary patient anxiety and burden.

Subsequently, though corrective measures were established through improvised kinetic measurements and a calibrator-based compensation for the interferents, the tests were also inconsistent when assessing the glomerular filtration rate (GFR), which is the gold standard estimate for diagnosing chronic renal failure [6,7]. Hence, many clinical laboratories have shifted to the enzymatic method which is inherently specific and hopes to produce reliable Cr measurements. While the enzymatic assays are less perturbed by the aforementioned substances, they are not spared completely from the interference complexities. The presence of IgM paraproteins and the infusions of different medications such as furosemide and catecholamines such as dopamine and dobutamine (DD) have been shown to negatively interfere with the enzymatic creatinine (E-Cr) assay, thus falsely lowering the measured sCr [8,9,10,11,12].

Previously, dopamine has been shown to interfere stoichiometrically with peroxidase-based tests that use 4-aminophenazone to form chromophore, whereas dobutamine interferes with all peroxidase-based tests regardless of 4-aminophenazone use [11]. DD can also either undergo more rapid oxidation than the indicator dyes, causing a depletion of the hydrogen peroxide required to oxidize the indicator chromophore or change the chromophore’s chemical stability [11,13,14]. There are several peroxidase-based tests currently utilized in routine testing, including E-sCr. Clinically, DD-based interference does impact the sCr measurement when blood is drawn from a central venous catheter (CVC) or peripherally inserted central venous catheter (PICC) while a catecholamine infusion is still running [9]. Thus, it is apparent that the patient- and practice-spawned interfering factors could diminish the accuracy of E-sCr measurement in selected contexts. Importantly, most Clinical Laboratory Improvement Amendments (CLIA)-certified laboratories operate in a ‘just right’ resource arrangement and will not have two different (methodology) assays for the same test parameter.

Of note, with the rising trends in the use of catecholamines, such as DD, to treat shock/hypotension and augment cardiac output, the degree of awareness that caregivers, including clinicians (decision-makers), nurses, and pharmacists, possess about the way these medications interfere with the E-sCr laboratory assay becomes pivotal. Furthermore, at the UT Southwestern Medical Center, we use enzymatic assays of sCr and have encountered multiple critical-care physician complaints and requests for retesting because of inconsistent sCr results, which led to the current quality improvement project. Currently, no data are available on whether the critical care providers are aware of this complexity. Given this, we sought to gauge their awareness of E-sCr interference by conducting a survey about their baseline knowledge of this problem and we also performed laboratory studies to assess for negative interference.

## 2. Materials and Methods

### 2.1. Study Population, Evaluation Period, and Method Details

We performed this study as a part of a quality assessment/quality improvement task based on our clinician complaints about unreliable creatinine values in patients undergoing DD infusions (low dose <5 mcg/kg/min). This specific purpose for the investigation did not involve any additional collection of samples and only used remnant samples after routine testing. We utilized sCr and other test results generated as part of the standard of care for the data analysis. The UTSW Institutional Review Board (IRB) waived consent and approved this study.

We performed all sample testing on the Roche Cobas c501 and c701 auto-analyzers in the core laboratory of our multi-specialty, tertiary care, university hospital. We utilized Roche Cobas enzymatic creatinine (E-Cr), the isotope dilution mass spectrometry (IDMS)-traceable assay for routine sCr testing, and the kinetic enzymatic assay using a coupled urease/glutamate dehydrogenase system for the determination of urea/BUN. Specifically, for this clinical investigation, we utilized kinetic colorimetric assays based on the Jaffe method as a predicate for the comparison of E-Cr results. The Jaffe method has no known interference from catecholamines [8]. All assays were performed within 24 h of collection by both methods in order to not introduce any sample stability-induced variability.

### 2.2. Cross-Sectional Assessment of sCr Results: PICC/CVC versus PV Draws

Initially, we compared the E-Cr results versus the Jaffe method using randomly selected (in no particular order) remnant blood samples that were available in the laboratory after completion of the routine testing. These blood samples were collected from patients on continuous DD infusions and included both CVC/PICC line- and PV-drawn samples. In our facility, qualified nurses perform all the subjects′ CVC/PICC collections and phlebotomists perform the PV collections. The acceptance criteria of sCr results were fixed as per the CLIA and Clinical Assessment Protocol (CAP) at an allowable error margin of 15% or 0.3 mg/dL (whichever is greater).

### 2.3. Longitudinal Assessment of sCr in Patients on DD Infusions

In this study, we also reviewed the longitudinal data of E-sCr levels reported in patients undergoing active DD infusions to identify any potential suspect sCr values (outliers). In parallel, we evaluated the longitudinal data of other renal markers, including BUN and potassium levels, in the same group of patients. The longitudinal assessment included only the CVC/PICC line samples, as a cross-sectional assessment did not show any significant interference in the PV samples. These cardiopulmonary patients did not have an arterial line placed for additional draws. For the longitudinal comparison of each patient, we included PICC line sample results from at least 6 time points reported over a 3-day period. Upon reviewing the longitudinal data of the sCr, BUN, and potassium levels in each patient for major dips and elevations along with the overall trend. If the overall sCr data did not correspond with BUN changes and with the clinical data, the values were deemed to be erratic. We then compared the inconsistent value to a mean of 4 immediate values (2 previous and 2 after values) of the same analyte. We considered the report to be significant if the deviation was greater than 30% (arbitrary threshold). For the calculation of a mean value for comparison, 2 or more subsequent suspect values were excluded, while their adjacent values were considered. We assessed potassium levels in parallel with sCr to rule out the dilution effect of a line draw.

### 2.4. Assessment of Care Provider’s Awareness about DD-Based Negative Interferences in E-sCr Assay

Our critical care team members, comprised of clinicians, nurses, and pharmacists directly involved in the management of patients on DD infusions, were invited to complete a detailed awareness survey. The survey questionnaire contained 9 distinct conceptual questions that could potentially predict the caregivers’ knowledge and awareness about DD-induced E-sCr assay interference. In particular, the questionnaire assessed these in three areas, including local/hospital system awareness (3 related questions), field-related knowledge (3 related questions), and personal factors (3 related questions). After obtaining the required permission from the relevant university hospital authorities and as a part of the quality assessment task, we administered this survey using “redcap”, which is a secure electronic data collection software application. To conduct our research without bias while administering the survey, an appeal was made to each participant not to share or discuss the questionnaire details with other team members.

## 3. Results

### 3.1. Survey Findings

Understanding the influence of critical care providers’ awareness of interference in laboratory assays has relevance to both the profession and patients. Thus, we sought to examine the preferred draw patterns and identify awareness about DD-induced interference in sCr assays among our critical care team members, including physicians, nurses, and pharmacists, by first leveraging their opinions and views given in the survey. The survey data were collected and a total of 141 members took part. In the first step of our analysis, we filtered the total participants to 118 based on their active involvement in prescribing and/or managing patients on DD infusions and on their completion of the full survey: this included 39 physicians, 56 nurses, and 23 pharmacists.

**Figure 1:**
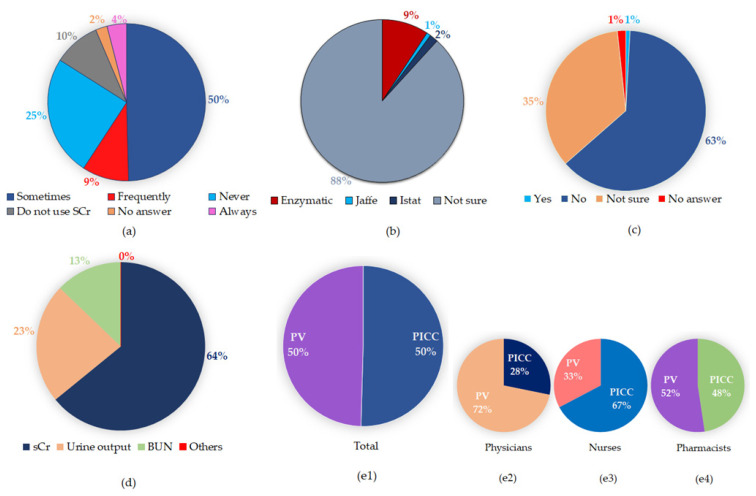
Survey findings. (**a**) Have you noticed erratic creatinine (Cr) results? (**b**) Which Cr assay is used in your main lab? (**c**) Are you aware of DD interference in E-sCr results? (**d**) What is the preferred means of monitoring renal function in critical care patients? (**e1**–**e4**) Preferred draw site in these patients. (**e1**) Total; (**e2**) physicians; (**e3**) nurses; and (**e4**) pharmacists. E-sCr: enzymatic serum creatinine; PV: peripheral vein; PICC: peripherally inserted central catheter.

The questions about whether the participant had noticed erratic sCr assay results and the type of sCr assay used were designed to map the critical care team members’ awareness and knowledge of their hospital laboratory-related practices. Nearly 63% (*n* = 79) of the respondents had noticed erratic creatinine results in their practice. Of this group, a majority of them had noticed erratic results sometimes (*n* = 62) and very few noticed it frequently or always. In the catecholamine-infused patients (Figure 1a), 25% of the caregivers had not noticed any questionable creatinine result. Among our critical care team members, 89% (*n* = 105 out of 118) of respondents were not aware of the specific creatinine method used in our main lab (Figure 1b). Only 11 out of the 118 participants correctly picked the E-sCr assay as the routine method used. However, most of them were also not aware of any pre-analytical steps that can impact the creatinine results. This shows a tremendous gap in both the knowledge and understanding of the local laboratory practice among our critical care group.

To explore the critical care groups’ field-related knowledge, the question related to DD interference in E-sCr assays was designed and probed. Figure 1c illustrates the results, and strikingly, it was found that the majority (82%) of the critical care team members were not aware of DD interference in the E-sCr assay. This response was equal among doctors, nurses, and pharmacists. This result indicates a lack of field-related awareness and suggests that some sort of educational intervention is essential to bring positive changes in knowledge and awareness about DD-induced E-sCr interference.

In any hospital setting—this includes the critical care unit of physicians, nurse practitioners, and pharmacists—while following an accepted medical practice, one can still remain different in one’s personal and clinical-practice characteristics and preferences. In probing for the preference of our critical care team members’ choice when using sCr as a surrogate for renal function, we observed a distribution of preferences among the survey participants. Only 65% of the survey participants preferred sCr for monitoring renal function. About 23% of them preferred urine output, and the remaining participants preferred BUN and phosphorus (Figure 1d). Notably, the choice of site for blood draws can induce spurious test results based on the contexts of duration, medication type, and erroneous handling, as has been seen in the case of PICC/CVC and PV line draw samples [9,15,16]. Thus, understanding the caregivers’ draw preferences is critical and will indirectly allow us to grasp their clinical judgment beyond ease of access. Our results show that there was an equal distribution of overall preference for PICC and PV blood draws among the critical care team members (Figure 1(e1)). In particular, 72% of the clinicians preferred PV over the PICC line draws (Figure 1(e2)). In contrast, 66% of the nurses reported that the PICC/CVC line was their preferred site of blood sample collection relative to PV (Figure 1(e3)). Pharmacists chose PICC/CVC line and PV collections equally (Figure 1(e4)). This result suggests that there was a stronger inclination among the nurses to use a PICC/CVC line when compared to the doctors and pharmacists in our critical care unit.

### 3.2. Comparison of Cross-Sectional PICC/CVC and PV Line sCr Results in DD-Infused Patients

A total of 139 routine blood samples were collected from critical care patients on continuous DD infusions. They were tested using E-sCr assays as part of the standard care and in no particular order they were randomly selected to be tested using the kinetic Jaffe method. This included 113 PICC/CVC line samples and 26 PV samples from 76 patients. Using Jaffe’s Cr results as a predicate and an acceptance criterion of 15% or 0.3 mg/dL for sCr, we found that 12% of PICC line sample results were unacceptable because they exceeded the allowable error (Table 1). In contrast, none of the PV draw results displayed error beyond the allowable limit, thus indicating the absence of interference.

### 3.3. Longitudinal Comparison of sCr and BUN Results in PICC Draws

To confirm our findings from the cross-sectional study, we further longitudinally assessed sCr values against the other renal function marker, blood urea nitrogen (BUN) routinely monitored in patients with PICC/CVC line samples. Longitudinal assessment excluded the PV samples as the PV cross-sectional analysis did not exhibit any interference. The total number of patients that had valid longitudinal data (a minimum of 6 data points spread over 3 days for each analyte) was 50. The average number of data points and days included in our longitudinal study population was about 18 points and 8 days. Twelve out of 50 patients (24%) showed a significant reduction in creatinine values that did not match with a corresponding decrease in BUN or potassium (K) values (Table 2). Out of 904 E-sCr results reviewed in the longitudinal study, 21 (2.3%) results had significant negative inference from DD-infusion.

**Figure 2:**
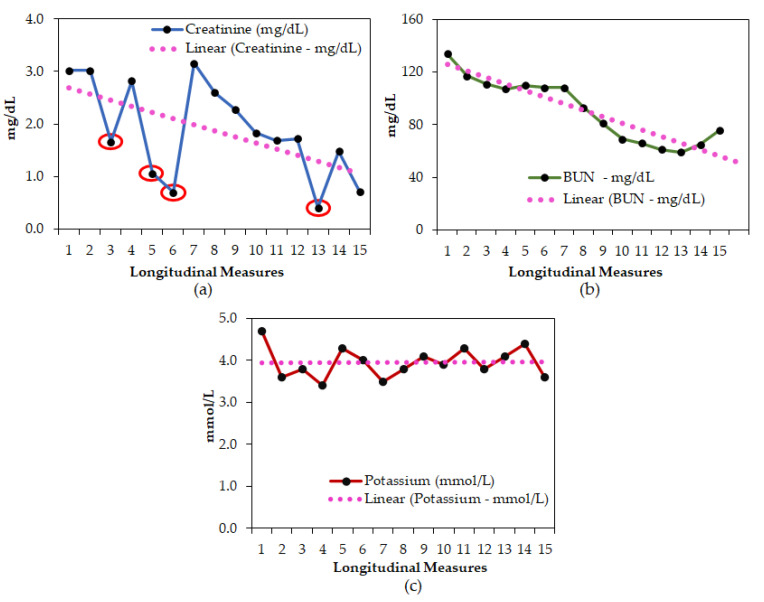
A representative longitudinal assessment of (**a**) sCr; (**b**) BUN; (**c**) K results from patient #1. Red circles in panel (**a**) indicate erroneous sCr results.

In our longitudinal comparison of the serial measurements of s-Cr versus BUN or K—for example, in “Patient 1”—we show that DD infusion has led to multiple discrepant sCr values (erroneously low on four different occasions—red circles; Figure 2a) with steady BUN levels (Figure 2b) and without a huge oscillation in K levels (Figure 2c). The other 11 patient samples with discrepant sCr values with relatively unchanged BUN and K levels were provided in the supplementary Figures S1–S6.

## 4. Discussion

The potential interference in E-sCr assays during DD infusions has been the subject of previous studies [8,9]. The approach described herein differs from the earlier studies [8,9] in that it combined the qualitative research inquiry (awareness survey) with the quantitative analysis (laboratory assessments/validations) to obtain a better understanding of how to approach error identification in sCr measurements. Thus, we believe that our real-time perspective of the critical care team in combination with the laboratory data balances the qualitative and quantitative research, and hence it could be effective in reinforcing action that creates a sustainable improvement at the individual laboratory level [17,18]. In addition, this is the first study to compare the rate of DD interference in both a cross-sectional and longitudinal format for DD-infused patients. The cross-sectional assessment of E-sCr showed that it is only the CVC/PICC line samples that encountered negative interference and not the PV draws. Although this is consistent with a previously published study that suggested that CVC/PICC line draws might interfere because the siphoning effect from the lumen infuses DD into the collection lumen [9], we observed that only 12% of the CVC/PICC line specimens showed negative interference. This suggests that while most of the nurses in our critical care facility are well-trained and conform to the standard operating procedure for the line draws, there is still room for improvement and a chance to make better use of valuable healthcare resources.

In our longitudinal assessment, the overall percentage of CVC/PICC line samples with discrepant creatinine results from the cross-sectional study was much lower than expected. However, the number of impacted patients (24%) was significantly higher in the longitudinal study. This direct comparison study shows that a longitudinal assessment of creatinine results is more useful when trying to understand the true impact of DD interference on E-Cr results. Serial measurements of s-Cr versus BUN and potassium in our longitudinal analysis clearly showed that DD infusion led to erroneously low sCr values on multiple occasions—for example, in “Patient 1”—whereas BUN and potassium levels were steady. At a time when the critical care team expects an immediate turnaround of tests and the insurance companies keenly seek to contain healthcare costs, any uncertainty or deviation in an individual sCr result, such as those observed, may complicate the authenticity of the results, thus tempting some towards a presumptive diagnosis that adds ambiguity to clinical decision making.

Another striking finding is that our survey analysis has identified three major challenges among our critical care colleagues pertaining to (i) the lack of general awareness about the local/hospital laboratory practices, (ii) the lack of specific knowledge (field literacy) about using PICC/CVC or PV lines and treatment-related assay interference (here, DD infusion-E-sCr assay), and (iii) misinformation that E-sCr assays are specific and not subjected to interference. While 63% (*n* = 79) of the critical care team members had noticed erratic s-Cr results at some point in their patients, a majority (82%) were not aware of DD interference in the E-sCr assay. More importantly, only 65% of the critical care professionals used sCr for monitoring renal function and the remaining 35% preferred other indices such as urine output, BUN, or phosphorus. The likely reason is that for some who have noticed erratic sCr values, all creatinine results are suspect and that compels them to rely on other markers. Regarding their preference in their choice of sampling, most preferred PICC/CVC draw as their method of sample collection likely due to convenience, ongoing traditions, and/or a decrease in the needle sticks associated with PV collection. In particular, the nurses at our critical care facility had a strong preference for PICC/CVC over the PV line when compared to doctors and pharmacists. However, in our case, the DD-based E-sCr interference was only observed in PICC/CVC draw and PV samples were unaffected. Thus, to avoid the consequences of faulty assessments of renal function in critically ill patients under such circumstances, it is essential to first deconstruct the misconceptions that all enzymatic assays are specific. As a pre-emptive measure, it is imperative to educate the nurses regarding the influence of E-sCr assays on DD infusion and to emphasize that they adhere to PV draws over PICC for sCr where possible. When PV is not likely, a meticulous and extensive flushing of the PICC/CVC lines are essential to remove the DD remnants loading onto the test sample. Incorporating this into the nurses’ education may bear significant influence in improving the potential of patient engagement and outcomes since in the critical care/hospital setting, nurses coordinate most of the patient-related cares and needs routinely in a face-to-face manner by the bedside. This provides them with a better platform to communicate with and educate the patients about the medications administered as well as the complex processes involving laboratory testing and sampling (draw) preference. Alternatively, laboratories could use the alkaline picrate method for assessing s-Cr in patients with DD infusions. However, when the in-house alternative option is practically prohibitive, it can be a ‘send-out’ to external vendors that use the appropriate sCr assay (not enzymatic assay). Beyond just normal and abnormal results being flagged, knowledge about DD-based interference on E-sCr assays can also be programmed to the clinical decision support system (CDSS) engine such as that it pushes out proper alerts and suggestions on a case-by-case basis during the results relay.

Our study has multiple limitations. (1) This was a quality improvement study, so we did not access patient-level data to evaluate the harm incurred from interference. While it is reasonable to assume that an inaccurate assessment of creatinine would interfere in clinical care and decision making, we do not have any directly sourced data to verify this claim. (2) While the predicate sCr method used herein suffers less from DD-based interference, this can also be insufficiently accurate owing to the other reactive components in the serum. Given that there is no ‘one-size fits all’ assay for sCr, a judicial and educated case-by-case approach is the best way to obtain precise results. (3) This study pertains to the assessment of critical care providers’ knowledge of sCr interference at our institution, so the results may not be generalizable to other scenarios/institutions. This study only provides a framework for future analogous studies in different or comparable formats.

## 5. Conclusions

In our study, we have identified a concerning knowledge gap in our clinical care practice about DD-derived interference in E-sCr assays. To alleviate this problem, a related educational intervention for the professionals and an embedding of the CDSS with automatic flagging for suitable reflex sCr testing either in-house or on ‘send-out’, whichever is applicable, could be valuable. This will eventually help formulate diagnostic accuracy and improve the standard of care in the select patients who require DD infusions.

## Figures and Tables

**Table 1:** Comparison of peripherally inserted central catheter (PICC) line and peripheral vein (PV) enzymatic serum creatinine (E-sCr) results using the kinetic Jaffe method as a predicate.

Blood Collection Site	Total Patients	Total Samples	E-sCr	
			Acceptable %	Not Acceptable %
PICC	*n* = 57	*n* = 113	88	12
PV	*n* = 20	*n* = 26	100	0

**Table 2:** Information of DD-infused patients with impacted E-sCr in longitudinal comparison with BUN or potassium results.

Blood Collection Site	Total Patients	Total Patients Impacted	Total Samples Tested	Total Samples Impacted
PICC	*n* = 50	12 (24%)	904	21 (2.3%)

## Data Availability

Due to ethical reasons, the data are not publicly available. However, upon request, the data presented in this study can be shared.

## Supplementary Materials

The following are available online at https://www.mdpi.com/article/10.3390/jcm10081689/s1, Figure S1: A longitudinal assessment of (a) E-sCr; (b) BUN; and (c) K in patients on DD infusions (patients 2 and 3), Figure S2: A longitudinal assessment of (a) E-sCr; (b) BUN; and (c) K in DD-infused patient samples (patients 4 and 5), Figure S3: A longitudinal comparison of (a) E-sCr; (b) BUN; and (c) K in patients on DD infusions (patients 6 and 7), Figure S4: Longitudinal analysis of (a) E-sCr; (b) BUN; and (c) K in DD-infused patient samples (patients 8 and 9), Figure S5: A longitudinal comparison of (a) E-sCr; (b) BUN; and (c) K in DD-infused patient samples (patients 10 and 11), Figure S6: Longitudinal assessment of (a) E-sCr; (b) BUN; and (c) K in patients on DD infusions (patient 12).
