# Supplementary material for: Variable Creatinine Levels in Critical Care Patients: A Concerning Knowledge Gap
Source: J Clin Med. 2021 Apr 15;10(8):1689. doi: 10.3390/jcm10081689 (PMC8071156; doi:10.3390/jcm10081689)
Supplement: Supplementary file 1 [file jcm-10-01689-s001.zip › jcm-1149738-supplementary/jcm-1149738-supplementary.pdf]

Supplementary materials

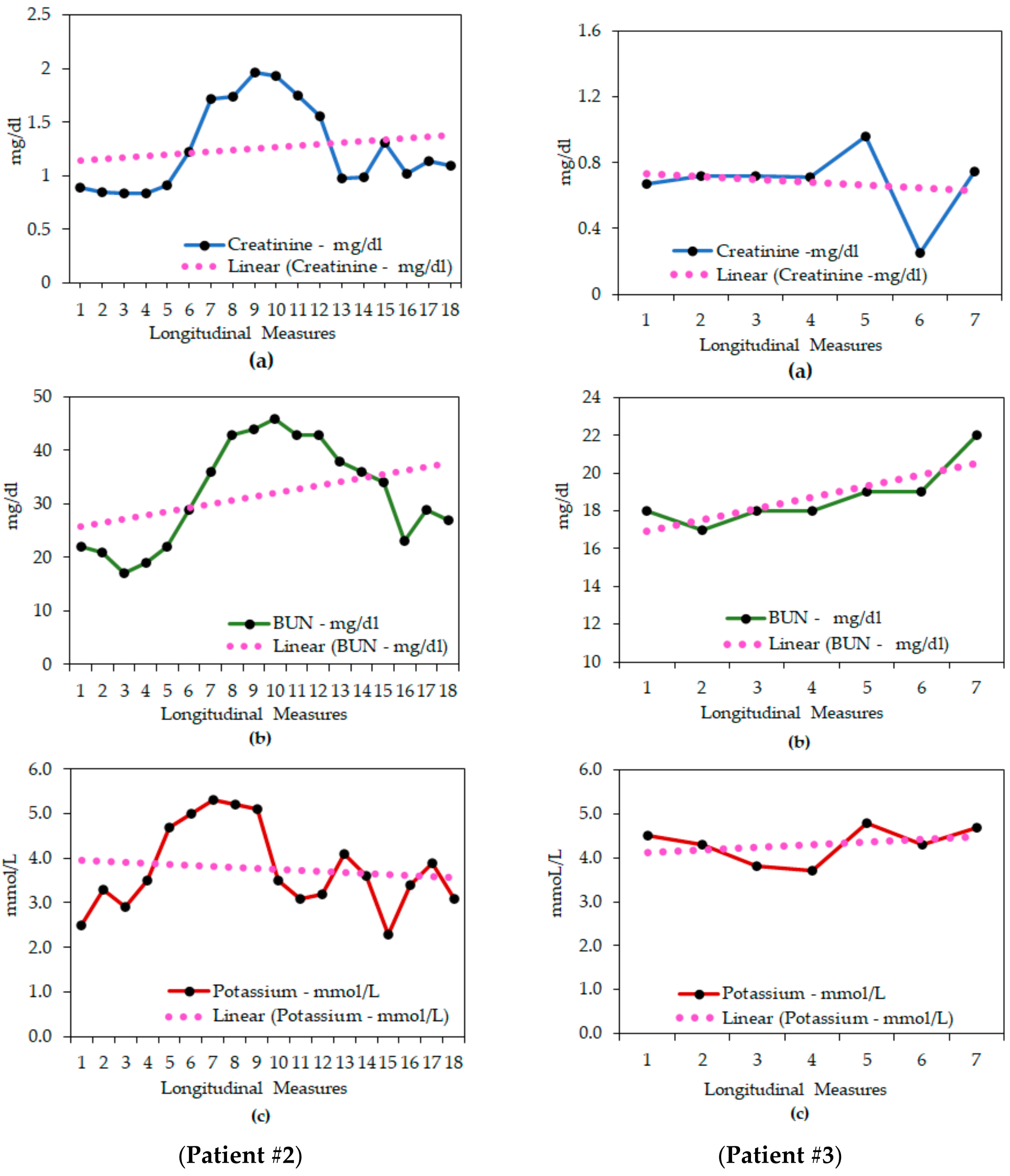

Figure S1. The longitudinal assessment of a) E-sCr; b) BUN; and c) Potassium in patients on DD infusions. The longitudinal assessment of a) E-sCr; b) BUN; and c) Potassium in 12 patients that had erratic E-sCr values following DD infusions (11 provided here and 1 as a representative in the manuscript).

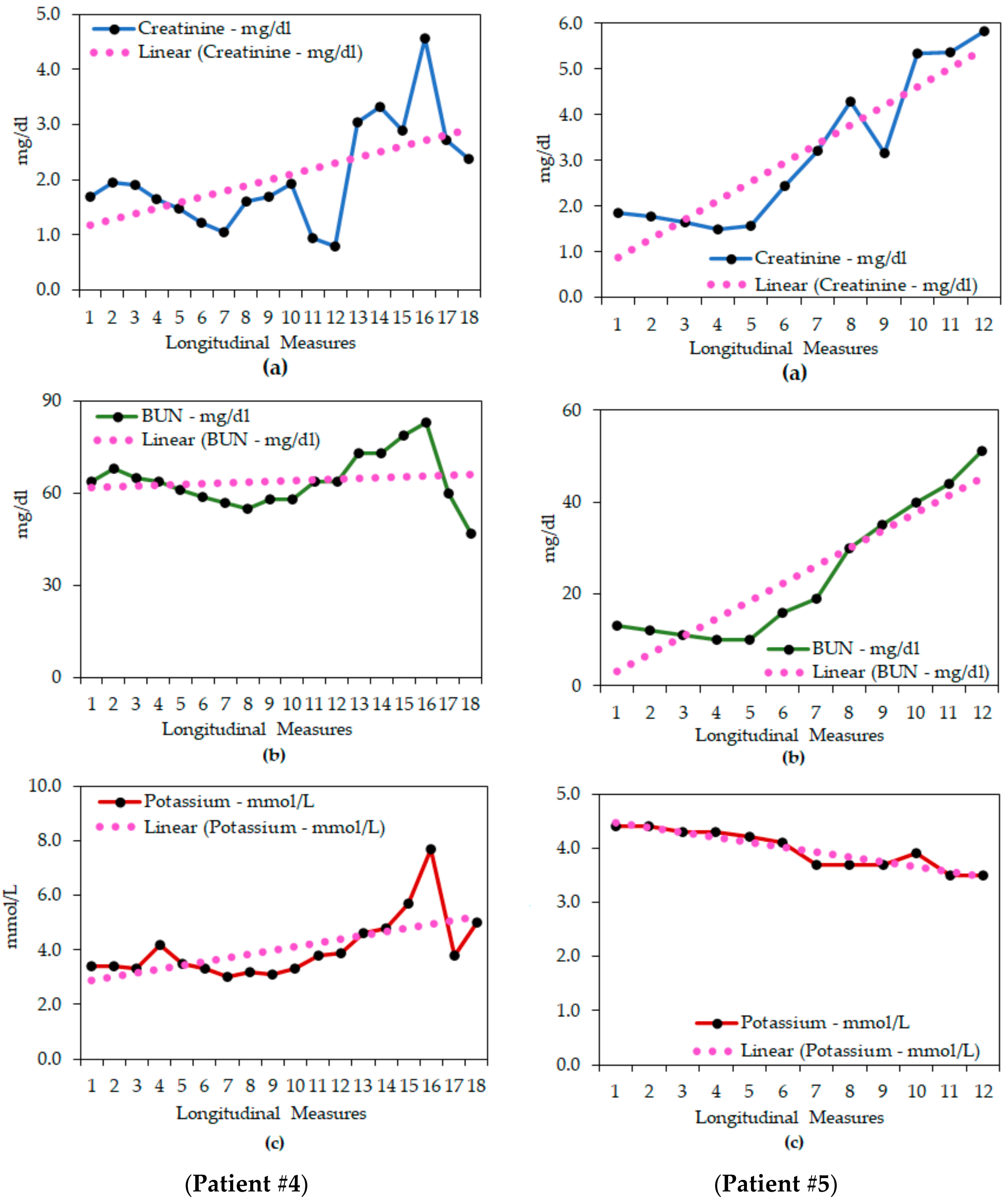

Figure S2. The longitudinal assessment of a) E-sCr; b) BUN; and c) Potassium in DD-infused patient samples.

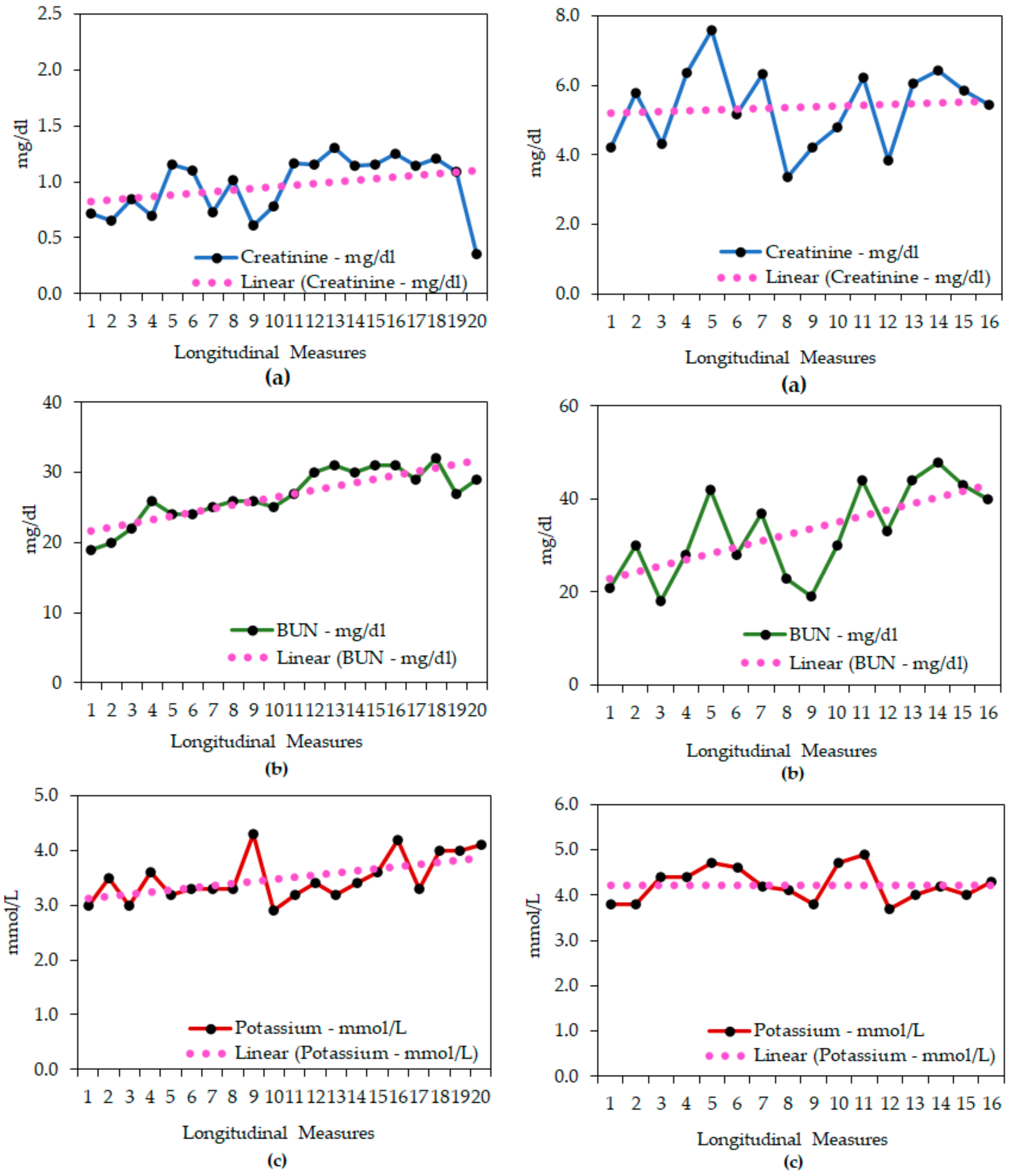

(**Patient** #6) (**Patient** #7)

Figure S3. The longitudinal assessment of a) E-sCr; b) BUN; and c) Potassium in patients following DD infusions.

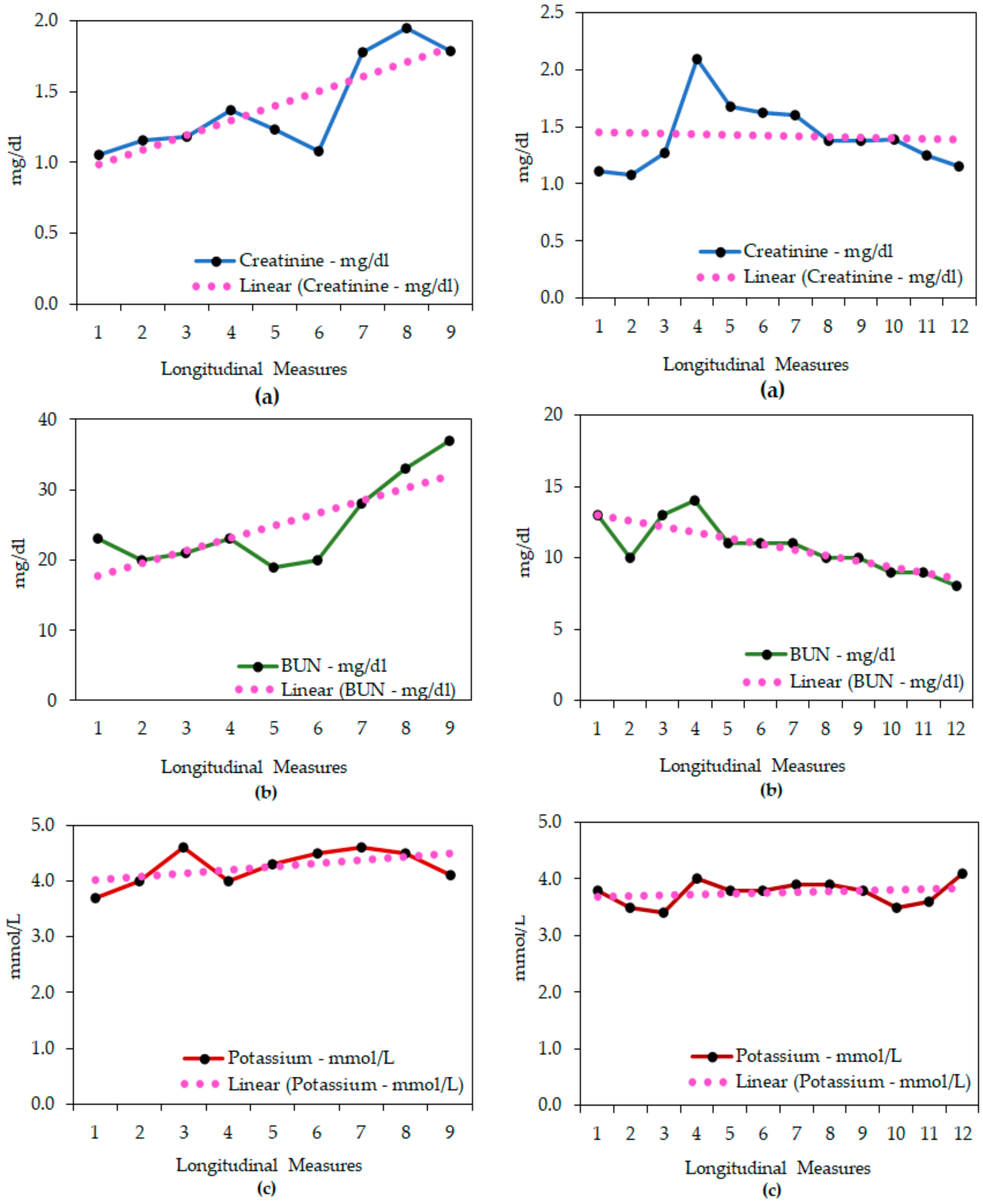

(Patient #8) (Patient #9)

Figure S4. Longitudinal analysis of a) E-sCr; b) BUN; and c) Potassium in DD-infused patient samples.

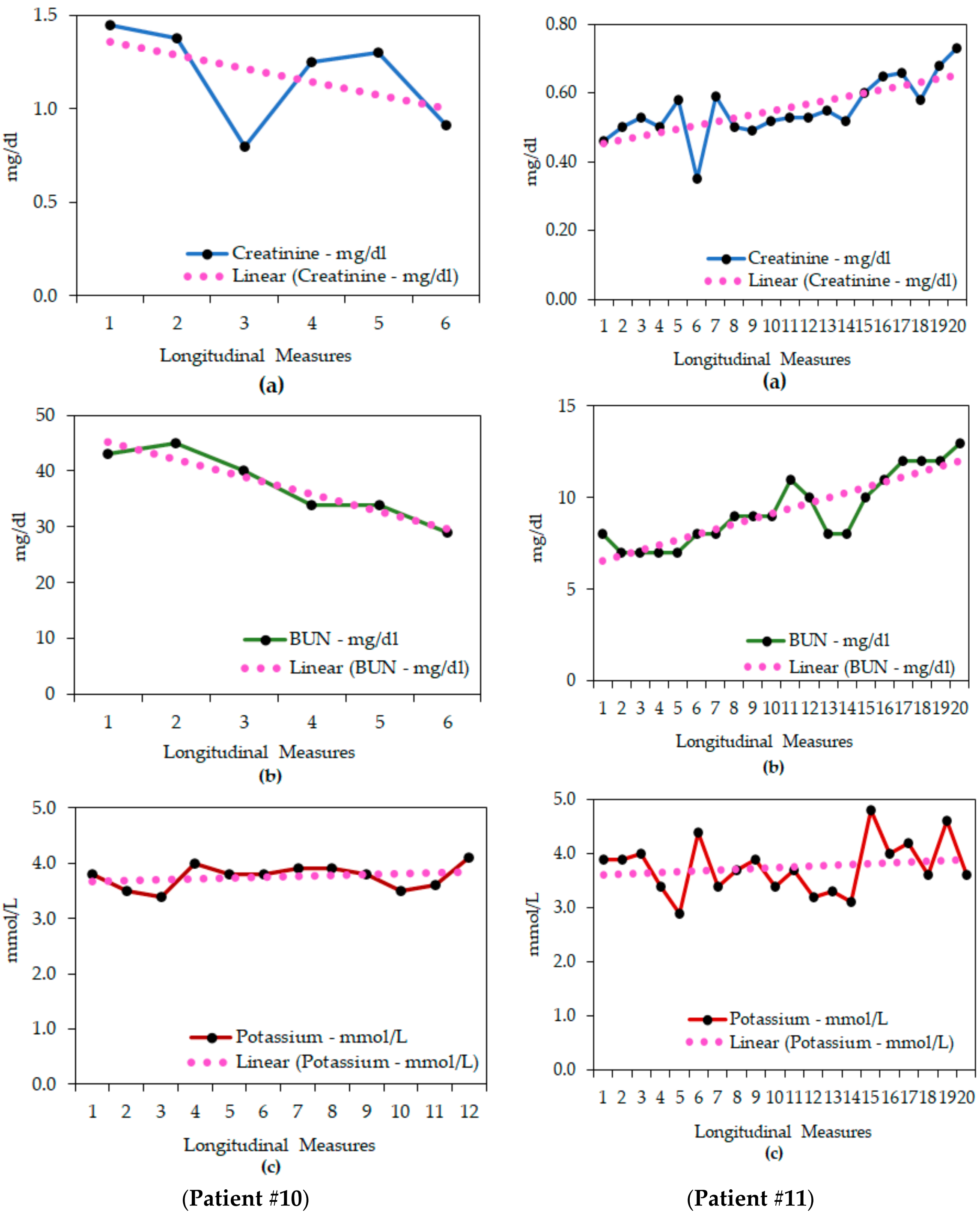

Figure S5. A longitudinal comparison of a) E-sCr; b) BUN; and c) Potassium in DD-infused patient samples.

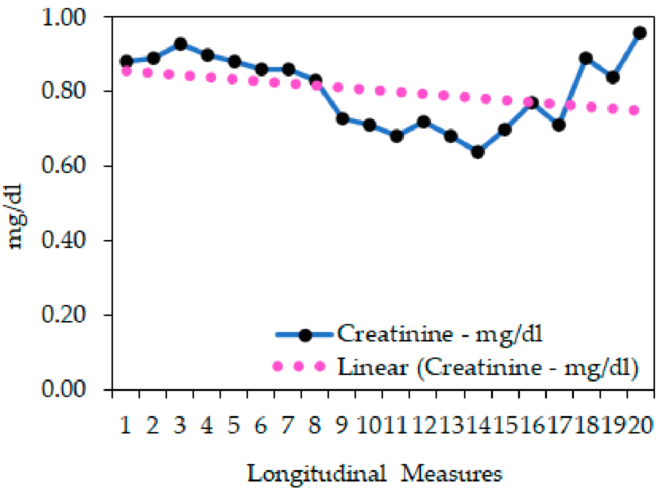

(a)

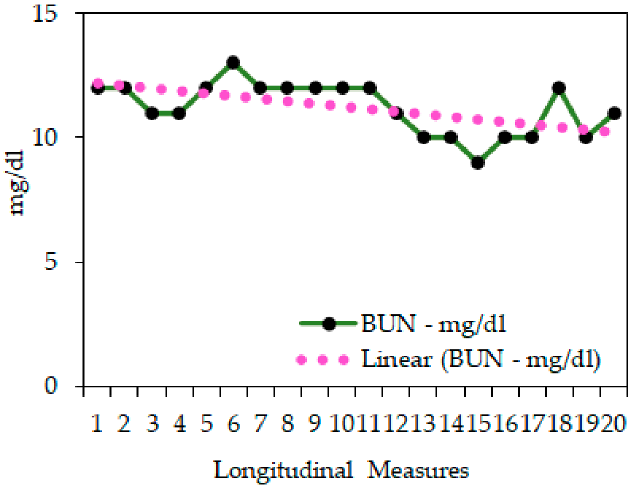

(b)

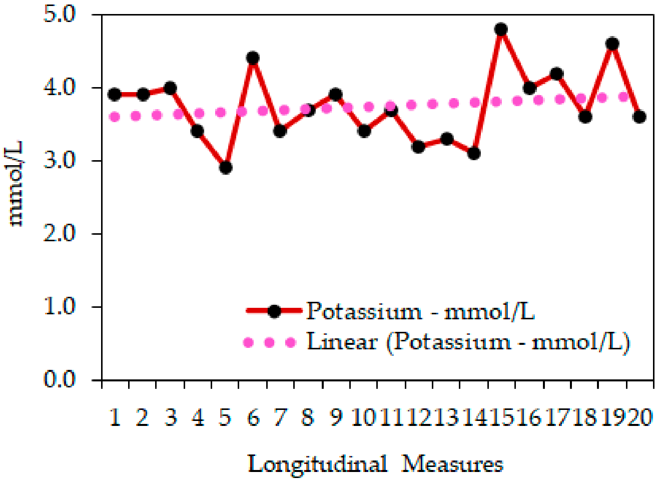

(c)

Figure S6. Longitudinal assessment of a) E-sCr; b) BUN; and c) Potassium in patients on DD infusions.
